# The relationship between attention control, emotion regulation and mental toughness among competitive female cricketers

**DOI:** 10.17159/2078-516X/2025/v37i1a20270

**Published:** 2025-06-15

**Authors:** SP Walker, SD Galhenage

**Affiliations:** Department of Psychology, University of the Free State, Bloemfontein, South Africa

**Keywords:** cricket, female, mental toughness, attention control, emotion regulation

## Abstract

**Background:**

Mental toughness is widely accepted as integral to consistent sporting performance. It has been proposed that attention control and emotion regulation contribute to mental toughness in several sporting populations, including elite cricketers. To date, there has not been any empirical attempt to test this proposed link among cricketers. Furthermore, no mental toughness research appears to have been conducted among female cricketers.

**Objectives:**

To determine whether attention control and emotion regulation predict mental toughness among high-level female cricketers in South Africa.

**Methods:**

High-level female cricketers (n=76) completed measures of attention control, emotion regulation and mental toughness. Descriptive statistics, reliability indexes and intercorrelations were calculated. A simple regression analysis was used to determine the contributions of attention control and emotion regulation in explaining the variance in the mental toughness of the participants.

**Results:**

Only the emotion regulation strategy of cognitive reappraisal demonstrated a positive correlation with mental toughness. In the regression model, attention control and emotion regulation accounted for 25% of the variance in the participants’ mental toughness scores (*R*^2^=0.250, *p*<0.001). However, cognitive reappraisal was the only psychological process to individually account for a statistically significant proportion of the variance in the female cricketers’ mental toughness scores (*β*=0.463, *p*<0.001).

**Conclusion:**

While a combination of attention control and emotion regulation appears to positively impact mental toughness among high-level female cricketers in South Africa, cognitive reappraisal seems to exert the strongest influence. Consequently, cognitive behavioural interventions aimed at fostering cognitive reappraisal skills could be beneficial for improving mental toughness in this population.

Mental toughness has been defined as “a personal capacity to produce consistently high levels of subjective or objective performance despite everyday challenges and stressors as well as significant adversity”.^[1]^ A growing body of research has identified mental toughness as central to success and consistency in various sporting and performance contexts.^[2,3]^ In addition, growing evidence suggests that mental toughness may be developed and trained via specific coaching methods and specialist psychological interventions.^[4]^ Despite the importance afforded to mental toughness as a performance-facilitating state and the investment in efforts to identify, develop and improve mental toughness, widespread consensus regarding the psychological processes that underpin mental toughness is still lacking.

Relatively little mental toughness research has been focused specifically on cricket. To date, two frameworks of mental toughness have been proposed for cricketers.^[5,6]^ Bull and colleagues employed qualitative interviews with twenty English test cricketers to gain insight into their perceptions of what constituted mentally tough character, attitudes and ways of thinking.^[5]^ Gucciardi and Gordon interviewed 11 Indian and five Australian current and former test cricketers to determine their views on what constitutes mentally tough behaviour and thinking.^[6]^ In addition to self-belief and determination to win, both studies identified attention control and emotion regulation as pivotal components in cricket-specific mental toughness.^[5,6]^ More recent research among elite batting coaches emphasised the importance of focused and flexible attention and well-developed emotion regulation skills to cricket batting expertise.^[7]^ A cricketer’s ability to effectively focus and shift their attention, thus, appears to be central to their mental toughness. Similarly, constructive regulation of emotional responses to internal states such as anxiety and individuals and events in the competitive environment seems facilitative of consistently high levels of performance.

Attention control is typically understood as the ability to effectively orient attention and resist distraction from competing stimuli to engage in goal-directed information processing.^[8]^ Attention control has traditionally been conceptualised as comprising focussed or sustained attention and divided attention.^[9]^ Sustained attention involves the ability to focus on a specific stimulus while limiting interference from task-irrelevant stimuli. Divided attention, also referred to as attention shifting, requires allocating attentional resources to multiple stimuli simultaneously and/or shifting focus between various stimuli. The ability to focus on important tasks and the effective shifting of attention have been positively associated with performance and mental toughness.^[1,5,6]^ Also, effective attention control has long been associated with reduced susceptibility to cognitive biases that typically underpin over-sensitivity to threat and anxiety.^[9]^ Attention control could thus be reasoned to promote mental toughness by facilitating task-specific focus and ameliorating the impact of cognitive biases associated with anxiety during high-pressure competition.

Emotion regulation has been conceptualised as how individuals modulate cognition and behaviour in response to the emotions they experience.^[10]^ Two emotion regulation strategies are said to be commonly employed, namely cognitive reappraisal and expressive suppression. Cognitive reappraisal involves reframing a situation that has the potential to elicit emotion in a manner that alters the emotional impact of the situation.^[10]^ For example, framing difficult batting conditions as an opportunity to test one’s technique, patience and character. Conversely, expressive suppression involves attempts to inhibit emotion-expressive behaviour.^[10]^ For instance, not letting on that one is nervous in the face of intimidating fast bowling. Cognitive reappraisal is generally preferable to expressive suppression as an emotion regulation strategy. Empirical evidence suggests that not only is expressive repression less effective at managing disruptive emotions, but it also tends to diminish the experience of positive emotions and lead to less favourable adaptive outcomes than cognitive reappraisal.^[11,12]^ It should be noted that the existing research on emotion regulation has been conducted primarily in community samples. Consequently, the applicability of these findings to high-level sporting contexts is currently unknown. Nonetheless, it could tentatively be speculated that emotion regulation, particularly cognitive reappraisal, may be positively associated with mental toughness.^[1,5,6]^

There have been increasing calls for sports psychology research in general and mental toughness research in particular to focus more on female athletes.^[13,14]^ Concern has been expressed that conceptualisations of performance-related constructs such as mental toughness tend to be viewed and understood primarily from masculine perspectives that may not reflect the experiences of female athletes.^[13,14]^ Given the lack of research on the psychological processes underpinning mental toughness among cricketers and the need for more mental toughness research among female athletes, the present study aimed to determine the role of attention control and emotion regulation in the mental toughness of high-level female cricketers in South Africa.

## Methods

### Participants

The General Human Research Ethics Committee at the University of the Free State approved the study ethics (UFS-HSD2023/0265/23). In addition, permission to recruit participants and collect data was obtained from Cricket South Africa. The management of the regional cricket governing bodies distributed information regarding the study to potential participants. Data were collected online. Players interested in participating in the study could follow a link to the informed consent form and electronic questionnaire. All participants were required to provide written informed consent before completing the questionnaire. Participants had access to the questionnaires for two months.

The final sample was comprised of 76 female cricketers. Participants ranged from 18 to 35 years of age (23.1±5.3 years), with 18-year-olds (26%), 19-year-olds (12%) and 20-year-olds (9%) most highly represented. Batters comprised 18% of the sample, while 29% identified as bowlers and 53% as all-rounders. For this study, wicketkeepers were classified as all-rounders. The majority (81%) of participants reported competing at the provincial level, 9% at under-19 level and 6% at the franchise level. Academy and development players accounted for 4% of the sample.

### Measurements

Mental toughness was measured using the Mental Toughness Index (MTI).^[1]^ The MTI is an eight-item self-report inventory that yields a unitary mental toughness score, with higher scores indicating increased levels of mental toughness. Response options that range from “False, 100% of the time” to “True, 100% of the time” are presented along a seven-point Likert-type scale. MTI has demonstrated good internal consistency (*ω*=0.80) in a sample of Australian undergraduate students.^[1]^

The 20-item Attentional Control Scale (ATTC) served as a measure of attention control.^[9]^ This self-report questionnaire provides response options along a four-point Likert-type scale, anchored by “almost never” and “always”. The ATTC yields a total score as well as two scale scores. Given that the current study focused on the role of both focused and divided attention in mental toughness, only the scale scores were used. The ATTC Focused Attention scale score (focussed/sustained attention) is calculated by summing the first nine items of the measure. In comparison, the sum of the other 11 items comprises the AATC Shifting Attention score (divided attention).^[9]^ In both instances, higher scores indicate better attention control. Cronbach’s alpha coefficients for both the ATTC Focused Attention (*α*=0.80) and ATTC Shifting Attention (*α*=0.65) scales indicate good to acceptable reliability in a sample of American undergraduate university students.^[9]^

The Emotion Regulation Questionnaire (ERQ) measured cognitive reappraisal and expressive suppression.^[10]^ The ERQ consists of 10 self-report items presented along a seven-point Likert-type scale with response options ranging from “strongly disagree” to “strongly agree”. Cognitive reappraisal is measured by summing responses across six items of the ERQ. The sum of responses across the remaining four items of the ERQ provides a measure of expressive suppression. Higher scores on the respective scales indicate more frequent use of cognitive reappraisal and/or suppression of expression as emotional regulation strategies. Internal consistency coefficients ranged from *α*=0.75 to *α*=0.82 for the Cognitive Reappraisal scale of the ERQ, and from *α*=0.68 to *α*=0.76 for the Expressive Suppression scale of the ERQ in a sample of undergraduate students in the USA.^[10]^

### Statistical analysis

Descriptive statistics, internal consistency coefficients and intercorrelations were calculated for all variables included in the study. Multiple regression analysis was employed to determine the extent to which divided attention, focussed attention, cognitive reappraisal and expressive suppression accounted for the variance in the mental toughness scores of the participants. Given the small sample size, a Bonferroni adjusted level of statistical significance of *α*=0.0125 (*α*=0.05/4) was set for all analyses.^[15]^ Analyses were conducted using the Statistical Package for the Social Sciences – Version 29 (SPSS-29).

## Results

The correlations between the ATTC Focussed Attention scale, ATTC Shifting Attention scale, ERQ Cognitive Reappraisal scale, ERQ Expressive Suppression scale and the MTI are displayed in Table 1. In addition, mean cores, standard deviations (SDs) and internal consistency coefficients are reported for each measure.

All measures, except for the ATTC Shifting Attention scale (*α*=0.56), meet the prescribed minimum level of acceptability for inclusion in psychological research (*α*=0.60).^[16]^ However, the decision was made to include the ATTC Shifting Attention scale in the analyses, as the construct has been proposed to be important in existing frameworks of mental toughness in cricket. ^[5,6]^

Indices of skewness and kurtosis do not indicate violations of the assumption of normality of the distribution of measures. Applying the Bonferroni adjusted level of significance (*α*=0.0125), the sample’s MTI scores demonstrated a statistically significant correlation with their scores on the ERQ Cognitive Reappraisal scale (*r*=0.52, *p*<0.001). Participants’ MTI scores were not significantly associated with their scores on the ATTC Focused Attention scale (*r*=0.18, *p*=0.131), the ATTC Shifting Attention scale (*r*=0.27, *p*=0.017) and the ERQ Expressive Suppression scale (*r*=0.21, *p*=0.068). Scores on the ATTC Shifting Attention scale and scores on the ATTC Focused Attention scale were significantly related (*r*=0.47, *p*<0.001). Statistically significant correlations were also found between the ERQ Cognitive Reappraisal scale scores and the ATTC Shifting Attention scale scores (*r*=0.33, *p*=0.003) and the ERQ Expressive suppression scale scores (*r*=0.30, *p*=0.003). All statistically significant (*α*≤0.0125) correlations reported in Table 1 are indicative of medium effect sizes (*r*=0.30 to *r*=0.49).^[16]^

Standard multiple regression analysis was employed to determine the extent to which focussed attention (ATTC-F), divided attention (ATTC-S), expressive suppression (ERQ-ES), and cognitive reappraisal (ERQ-CR) predict MTI among competitive female cricketers. Preliminary analyses indicated no violations of normality, linearity, multicollinearity and homoscedasticity assumptions. Consequently, the regression analysis was conducted as stated above.

**Fig. 1:**
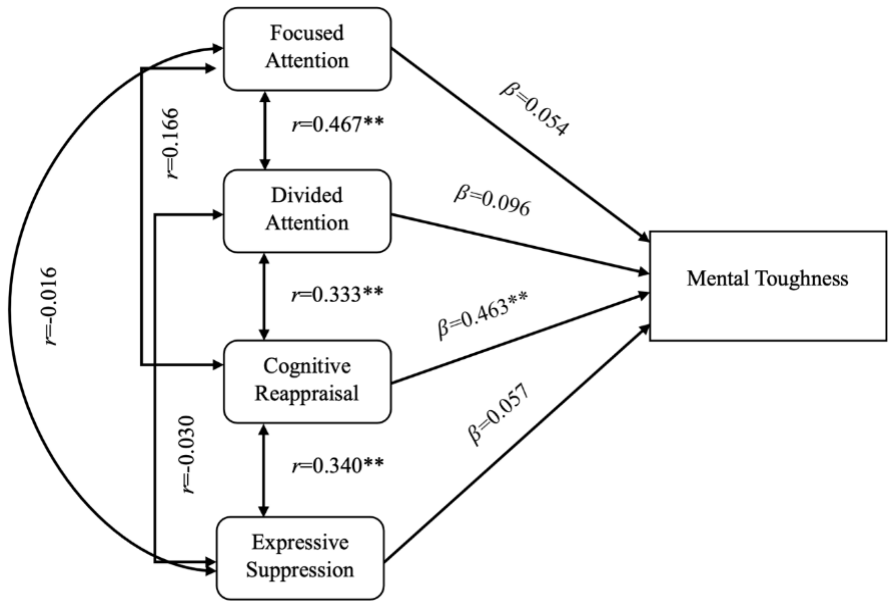
Regression model with focused attention, divided attention, cognitive reappraisal and expressive suppression as predictors of MT. ** indicates p<0.01.

The results of the regression analysis are reported in Table 2. The full regression model (combination of focussed attention, divided attention, cognitive reappraisal and expressive suppression) accounted for a statistically significant proportion of the variance in the sample’s MTI scores (*F*^(4,71)^=7.250, *p*<0.001). Despite the full regression model accounting for 25% of the variance in the female cricketer’s MTI scores (*R*^2^=0.250, *p*<0.001), only the emotion regulation strategy of cognitive reappraisal (*β*=0.463, *p*<0.001) emerged as a statistically significant independent predictor of the participants’ MT. This coefficient indicates a medium effect size (*β*=0.30 to *β*=0.49).^[17]^ The interactions between MT, focussed attention, divided attention, cognitive reappraisal and expressive suppression are displayed in Figure 1.

## Discussion

The current study addressed the lack of empirical research on the affective and cognitive processes associated with mental toughness among cricketers. The extent to which these constructs relate to the mental toughness of high-level female South African cricketers is of particular interest.

Mental toughness was significantly correlated with cognitive reappraisal. This finding aligns with theoretical assumptions that athletes’ ability to regulate their emotions effectively is a core component of mental toughness.^[1,3,4]^ Furthermore, this result is in keeping with the emphasis on emotion regulation in cricket-specific models of mental toughness.^[5,6]^ Qualitative studies of mental toughness among elite male cricketers also support the link between mental toughness and effective emotion regulation.^[5–7]^ The ability to cognitively reframe situations and events in such a manner as to modulate the emotions experienced during competition appears to be directly linked to mental toughness among high-level female South African cricketers. Conversely, attempting to modulate or regulate emotions by suppressing affect and/or inhibiting behaviours associated with the experience of emotion appears not directly associated with mental toughness in this population. The differing effect of cognitive reappraisal on mental toughness compared to the impact of expressive suppression on mental toughness corresponds to research conducted amongst university students and community samples.^[10–12]^ Consistent with findings from these populations, cognitive reappraisal among participants in the current study is linked to positive outcomes and mental toughness.^[10–12]^ Moreover, the current study indicates that expressive suppression is not associated with adaptive outcomes as in student and community samples.^[10,12]^ While high-level female cricketers are not directly comparable to university students, the associations between different emotion regulation strategies and adaptive outcomes appear consistent across these populations.

Neither the capacity to focus attention nor the ability to shift attention effectively were significantly associated with mental toughness in the current study. These findings contradict the existing theoretical perspectives on the role of attention control in mental toughness in general and cricket.^[1,3–7]^ There are several possible explanations for this discrepancy. First, the measure of attention used in this study was not specifically designed for sporting contexts, nor has it been normed on athlete samples. Second, the ATTC Focused Attention scale demonstrated suboptimum internal consistency in the current sample. Furthermore, existing reports of the scale’s internal consistency are in the lower threshold of acceptability.^[9,16]^ The development of sport-specific attention and attention control measures would provide clarity on whether the findings reported above are due to measurement error or some similar anomaly. Finally, it has been suggested that the relationship between attention control and performance may not be linear.^[8]^ Attention control might have an orienting function that enables improved emotion regulation, which is associated with mental toughness.^[8,9]^ Alternatively, cognitive reappraisal may lead to a more specific focus of attention, thereby enhancing mental toughness and performance.^[8,13]^ Consequently, attention control may act as a modulator of the relationship between emotion regulation and mental toughness. In this case, the relationship between attention control and mental toughness may not be direct, as implied by certain theoretical frameworks of mental toughness, but could be mediated or moderated by other cognitive-affective factors variables.^[4–6]^

Despite attention control and expressive suppression not demonstrating significant independent correlations with mental toughness in the current study, the combination of both components of attention control and both emotion regulation strategies accounted for a statistically significant proportion of the variance in the samples’ mental toughness scores. This finding appears to broadly support the notion that attention control and emotion regulation are, in some combination, positively associated with mental toughness.^[1,3–7]^ However, only cognitive reappraisal demonstrated a statistically significant independent relationship to mental toughness among the participants in the present study. This finding seems to support existing theoretical claims and empirical evidence that mental toughness is associated with the ability to reframe situations and events in a manner that serves to modulate emotional experience in such a manner as to facilitate performance.^[2,4–7,13]^ As mentioned above, the contribution of attention control and expressive suppression to mental toughness among the participants in this study may not be direct. While cognitive reappraisal directly impacts mental toughness, the other variables included in the study may influence mental toughness via cognitive reappraisal or vice versa.

Additionally, variables not included in the current study, such as personality traits, individual temperament, other cognitive-affective processes, competitive experience and level of competition, may be directly predictive of mental toughness and/or interact with attention control and emotion regulation to foster mental toughness.^[3–6,13]^ Nevertheless, cognitive reappraisal does appear to be an important component in the mental toughness of high-level female South African cricketers. The contributions of expressive suppression and attention control are less clearly understood at present.

### Limitations

The current study utilised a small sample of female cricketers. Consequently, the generalisability of the findings is quite limited. Nevertheless, the study represents an initial step toward a better understanding of mental toughness within this population. This study needs to be replicated in a larger and more representative sample. In addition, the size of the current sample prohibited exploring the impact of the level of competition and playing role on mental toughness, as well as on the relationship between attention control, emotion regulation and mental toughness. Additional research is required in this regard. The current study also used a cross-sectional correlational design. As a result, no conclusions can be drawn regarding possible causal relationships between the study variables. Longitudinal research would thus be beneficial in clarifying possible causal relationships and highlighting developmental and context-dependant (e.g., training versus competition) variations concerning cognitive-affective processes and mental toughness.

Given the relatively limited local research literature in sports psychology, none of the instruments used in the study have been normed on South African populations. However, except for the ATTC Focused Attention scale, these measures demonstrated acceptable to good reliability in the study sample. Notwithstanding, the development or adaptation of sport-specific measures of attention control and emotion regulation within the local context would benefit the field of sports psychology in general.

This study investigated the interaction between mental toughness and two cognitive-affective processes. Additional research is required to determine the contribution of individual differences, life experiences, resilience and numerous other factors to the mental toughness of female cricketers. Similarly, future research should test the possible mediating and moderating effect of attention and emotion regulation and other variables on mental toughness. Finally, small-scale qualitative studies would prove useful in developing theoretical models of mental toughness specific to this population.

## Conclusion

Attention control and emotion regulation are both associated with mental toughness among high-level female cricketers in South Africa. This supports existing frameworks of mental toughness in cricket. However, cognitive reappraisal seems to be the only cognitive-affective process that independently influences mental toughness in this group. Practically, developing cognitive reappraisal skills through cognitive-behavioural interventions appears beneficial for enhancing female cricketers’ mental toughness. The effectiveness of specific cognitive-behavioural interventions should be investigated further.

## Figures and Tables

**Table 1:** Correlations, reliability coefficients and descriptive statistics for the study variables (n=76)

	MTI	ATTC-F	ATTC-S	ERQ-CR	ERQ-ES

MTI	-	0.18	0.27*	0.52**	0.21
ATTC-F		-	0.47**	0.17	−0.02
ATTC-S			-	0.33**	−0.03
ERQ-CR				-	0.34**
*α*	0.88	0.70	0.56	0.74	0.72
M	42.7	23.5	29.1	28.8	17.6
SD	±9.0	±4.5	±4.2	±6.3	±5.7
Skewness	−1.68	−0.43	0.05	−0.60	−0.47
Kurtosis	3.60	−0.31	0.38	0.58	−0.002

** indicates p<0.01;

* indicates p<0.05.

M, Mean; SD, Standard Deviation; MTI, Mental Toughness Index; ATTC-F, Attention Control Focused Attention scale; ATTC-S, Attention Control Shifting Attention scale; ERQ-CR, Emotion Regulation Questionnaire Cognitive Reappraisal scale; ERQ-ES, Emotion Regulation Questionnaire Expressive Suppression scale.

**Table 2:** Model summary of regression analysis by blocks of attention control and emotion regulation variables predicting mental toughness (n=76)

Predictors	Adjusted R^2^	Δ R^2^	*β*	95% CI

**Mental toughness**				

*Step 1*				

ATTC Focused Attention	0.018	0.031	0.175	[−0.105, 0.798]

*Step 2*				

ATTC Focused Attention	0.053	0.047	0.060	[−0.383, 0.621]
ATTC Shifting Attention			0.246	[−0.016, 1.066]

*Step 3*				

ATTC Focused Attention	0.258	0.209	0.054	[−0.338, 0.550]
ATTC Shifting Attention			0.087	[−0.314, 0.688]
ERQ Cognitive Reappraisal			0.485**	[0.391, 0.990]

*Step 4*				

ATTC Focused Attention	0.250	0.003	0.054	[−0.340, 0.553]
ATTC Shifting Attention			0.096	[−0.303, 0.715]
ERQ Cognitive Reappraisal			0.463**	[0.335, 0.983]
ERQ Expressive Suppression			0.057	[−0.249, 0.429]

** indicates p<0.01
